# Effects of ultrasonic vibration on 3D printing of polylactic acid/akermanite nanocomposite scaffolds

**DOI:** 10.1016/j.heliyon.2024.e39240

**Published:** 2024-10-11

**Authors:** Mohammad Khodaei, Hamed Razavi, Hamed Nosrati

**Affiliations:** a Materials Engineering Group, Golpayegan College of Engineering, Isfahan University of Technology, Golpayegan, 87717-67498, Iran; b Mechanical Engineering Group, Golpayegan College of Engineering, Isfahan University of Technology, Golpayegan, 87717-67498, Iran; c Biosensor Research Center, Isfahan University of Medical Sciences, Isfahan, Iran

**Keywords:** 3D printing, Nozzle clogging, Ultrasonic vibration, Nanocomposite scaffolds, Polylactic acid, Akermanite

## Abstract

The mechanical properties of 3D-printed scaffolds for load-bearing implantation are crucial. Although the addition of nanoparticles to polymeric scaffolds can improve their mechanical and biological properties, due to certain limitations in printability, high amounts of reinforcement cannot be used. Therefore, in this study, an attempt was made to use ultrasonic vibration to inhibit nozzle clogging during fused filament fabrication (FFF) of polylactic acid (PLA) scaffolds containing 0, 20, and 40 wt% akermanite (Ak). Nozzle clogging happened during the conventional 3D printing of PLA-40 wt%Ak, while that did not occur during ultrasonic-assisted 3D printing of PLA-40 wt%Ak. Results of X-ray diffraction (XRD) analysis and scanning electron microscopy (SEM) indicated that applying ultrasonic vibration had no negative effect on the phases and morphology of the scaffolds. The results obtained by the compression test indicated that applying ultrasonic during 3D printing resulted in almost 27 % increment of elastic modulus and almost 25 % increase of the compressive strength of the scaffolds. As the conclusion, this study highlights the effectiveness of ultrasonic-assisted 3D printing in producing nanocomposite scaffolds with significantly higher nanoparticle loadings, as compared to conventional printing methods. By utilizing ultrasonic vibration during the printing process, the study showcases the possibility of overcoming viscosity limitations and optimizing the mechanical performance of scaffolds for various biomedical applications, including bone tissue engineering.

## Statement of significance

This study demonstrates the potential of using ultrasonic vibration to overcome a key limitation in the extrusion-based 3D printing of composite scaffolds - nozzle clogging during the printing of high-viscosity bioinks or those containing high amounts of reinforcement particles. The ability to print composites with higher nanoparticle content allows for the fabrication of scaffolds with enhanced mechanical properties for applications requiring load-bearing capability, such as bone tissue engineering. By applying ultrasonic vibrations to the printing nozzle, researchers can develop 3D-printed polylactic acid scaffolds reinforced with up to 40 % akermanite nanoparticles without any nozzle clogging issues.

## 1 Introduction

Additive manufacturing techniques have revolutionized the field of tissue engineering by enabling the fabrication of customized shaped grafts, scaffolds, and implants. Among these techniques, extrusion-based 3D printing has garnered considerable attention owing to its versatility and wide range of applications, notably in load-bearing implantation, where emphasis is placed on the mechanical properties of scaffolds [1]. Various techniques can be employed to enhance the mechanical properties of 3D-printed scaffolds. For instance, ultrasonic vibration can facilitate defect-free and continuous material injection and extrusion, thereby improving the overall quality of the printed scaffolds [2,3]. Extrusion-based 3D printing can be divided from two points of view. In terms of the type and state of the material that is printed, it is divided into fused filament fabrication (FFF), gel printing and direct ink writing (DIW) [4]. From the material extrusion mechanism point of view, it can be classified into three types: piston-driven, screw-driven, and pneumatic-driven devices [5,6]. The extrusion-based 3D printing technique is rapidly developing for fabricating tissue engineering scaffolds, due to some advantages such as fastness and inexpensiveness, processability for a wide range of materials such as polymeric matrix biocomposites and bioceramics, and appropriate resolution. This versatility allows researchers to tailor the properties of the printed scaffolds to meet specific requirements for a wide range of tissue engineering applications. 3D printers can print complex geometries of a wide range of biocomposites, without the need for initial filaments. For instance, the successful incorporation of nano-bioceramic particles, such as β-tricalcium phosphate (β-TCP) [7], hydroxyapatite [8,9], and bioglass [8], into polymers, such as polylactic acid (PLA) [9,10], polycaprolactone (PCL) [7], and polyglycolic acid (PGA) [11], has been reported to develop biocompatible and bioactive scaffolds with appropriate mechanical properties. It has been reported that the mechanical properties of 3D-printed scaffolds are significantly improved by increasing the content of reinforcement. Chen et al. added β-TCP from 5 to 20 wt% to poly vinyl alcohol (PVA) and printed the scaffold using FDM. They reported that the mechanical properties of composite scaffolds were significantly enhanced by increasing the β-TCP content up to 20 wt% [12]. They did not, however, report the effect of the higher β-TCP content.

While extrusion-based 3D printing offers numerous advantages, it faces certain challenges. One major issue encountered during the printing process is nozzle clogging, particularly when printing highly viscous materials or those with high reinforcement particle content [13]. The viscosity of bioinks that can be printed by extrusion bioprinting has been reported to range from 6 to 30 (× 10^7^) Pa.s [5]. The higher viscosities of the ink result in the scaffold retaining its shape after printing, but the possibility of nozzle clogging increases. To tackle this problem, some techniques such as using spherical particles as reinforcement [14], increasing the extrusion pressure [13], and applying plasticizers as lubricant materials [15] have been proposed. However, none of these techniques has been proved to be effective enough, thus highlighting the need for novel alternative approaches to address the problem of nozzle clogging during the printing process. Recently, some researchers have used other techniques, such as electrical field-assisted 3D printing or electro-melt writing (EMR) and ultrasonic-assisted 3D printing, to overcome nozzle clogging during the 3D printing of highly viscous materials [13,16,17].

Since the initial discovery and investigation of high-power ultrasonic vibrations by Blaha and Langenecker in 1955 [18], researchers have proposed ultrasonic potential to significantly enhance the efficiency of manufacturing processes and engineering materials. The beneficial effects of ultrasonic vibrations can be categorized into “volume effects,” which include a reduction in forming forces [[19], [20], [21]], a decrease in flow stresses [18,22], an increase in forming limits [23,24], and material heating [25,26]. These effects are documented in various studies. Additionally, “surface effects” encompass a reduction in contact friction [[27], [28], [29]] and an enhancement of surface quality [30]. The literature suggests several mechanisms that may contribute to the temporary softening of materials, including the absorption of ultrasonic energy, which facilitates dislocation movement on slip planes (often referred to as the potential-well hypothesis or acoustic softening) [[31], [32], [33], [34]], an increase in equivalent stress resulting from the superposition of ultrasonic stress component and conventional forming stresses [35,36], ultrasonic-induced temperature elevation [25,37], and a reduction in friction [27,[38], [39], [40]]. However, no single mechanism sufficiently accounts for the observed stress reduction or material softening, leading to the prevailing scientific consensus that a combination of these mechanisms is responsible [41,42]. Applying ultrasonic vibrations during molding of the porous materials in powder metallurgy process [43,44] and the flow of viscous fluids [45,46] or fluids containing nano/micro/macro particles [[47], [48], [49]] has been shown to significantly improve the process by reducing particles interlocking. For example, Quader et al. reported that by applying ultrasonic treatment to FDM 3D-printed PLA parts, the surface finish and the mechanical properties were improved [50]. Wu et al. also applied ultrasonic treatment to the bed (substrate) of a 3D printer, stating that the mechanical properties of the printed parts were improved significantly [51]. The most significant challenge in composite 3D printing is nozzle clogging. Therefore, in this research, we aimed to maximize the reinforcement content by employing ultrasonic vibrations on the nozzle of an extrusion-based 3D printer.

## 2 Materials and methods

### 2.1 Composite 3D printing

Different PLA-Akermanite composites (containing 0, 20 and 40 wt % Ak, hereafter named PLA, PLA-20Ak and PLA-40Ak, respectively) were prepared by dispersing akermanite (Ca_2_Mg [Si_2_O_7_], particle size<100 nm, purchased from Chakad Sanat Spadan Co., Iran) and dissolving PLA (Mw: 60,000, Merck Co., CAS No.: 26,100-51-6) in chloroform (Merck Co., CAS No.: 102,445), as reported previously [52]. After that, the composites were dried in an oven at 60 °C for 24 h; the dried composite was then fed to the printer dispenser barrel and heated to 200 °C. After melting, the material was extruded from a 600-μm nozzle by applying the pneumatic pressure of 2 bar. Nozzle movement at the speed of 10 mm/s was controlled by a 3D printer (Chakad, CSS1, Iran).

PLA-Ak composites (including different amounts of akermanite of 0, 10, 20, and 30 wt%) were 3D printed using a conventional extrusion base 3D printer, as done in our previous study [52]; however, in this study, the effect of ultrasonic vibration on the printability of PLA-Ak composite was investigated.

The application of ultrasonic vibrations on the nozzle of the 3D printer should be such that the maximum vibrations are focused and applied on the melt coming out of the nozzle. So, in this research, the horn tip was not fixed or welded to the nozzle, and it only touched the outer wall of the nozzle; this caused the nozzle and the melt inside to vibrate simultaneously. In this method, the harmonic impacts applied to the outer wall of the nozzle lead to the vibration of the melt exiting the nozzle, without the vibration system being affected by the size or material of which the nozzle is made.

### 2.2 Characterization

#### 2.2.1 Phases analysis

X-ray diffraction analysis (XRD: Philips X'Pert-MPD, Netherlands) was performed to identify the phases in the samples. X-ray was then generated using a Cu-Kα lamp with the wavelength of 1.54060 Å at 40 kV, with the rate of

2θ = 1°·min^−1^ in the range of 2θ = 10°–70°.

#### 2.2.2 Microscopic observation

After camera imaging, microstructural studies using scanning electron microscopy (SEM) of the structure of the scaffolds and powder were investigated by Scanning Electron Microscopy (SEM: Philips XL30) and ZEISS, respectively, after gold coating.

#### 2.2.3 Compressive strength

Measurement of compressive strength was done to assess the mechanical properties of the composite scaffolds; so, the compression test, according to the ASTM F2150-19 standard, with a cross-head speed of 0.5 mm/min, was conducted on the specimens with the dimensions of 10 × 10 × 10 mm^3^ at the ambient temperature. A universal testing machine (UTM: HOUNSFIELD: H30KS, UK) was then used for this evaluation and the stress-strain engineering curves of the specimens were drawn and the necessary information was extracted. This test was repeated 3 times for each sample; the mean values have been reported.

## 3 Results and discussion

### 3.1 Akermanite powder

**Fig. 1:**
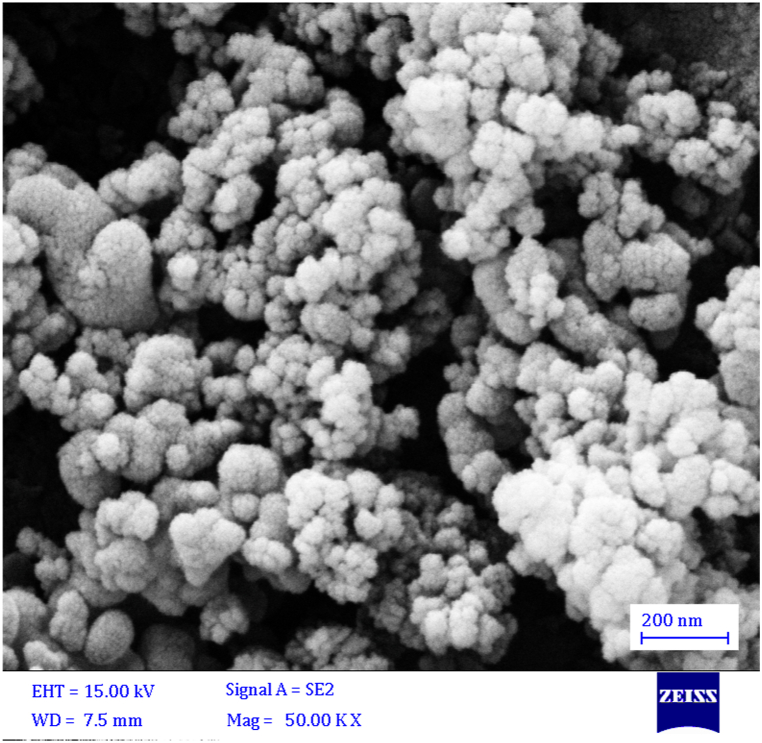
The SEM image of the used akermanite powder.

The SEM image of akermanite powder is presented in Fig. 1. Evidently, the particle size of akermanite was smaller than 100 nm. The particles were semispherical in shape and agglomerated.

### 3.2 Phases analysis

**Fig. 2:**
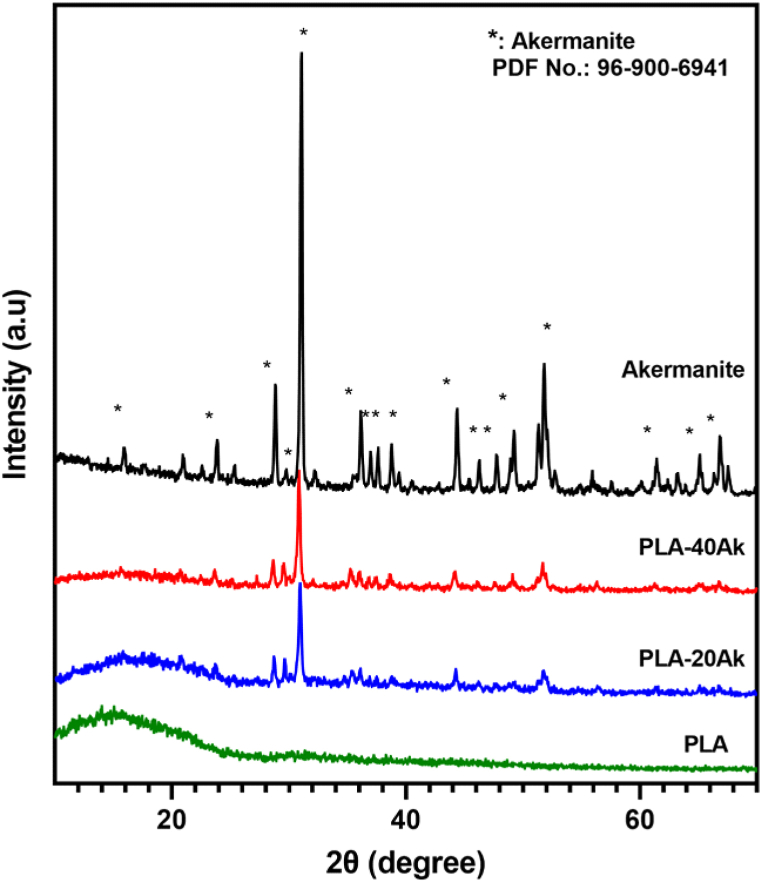
XRD graphs of pure PLA, PLA-20Ak, PLA-40Ak composites, and akermanite powder.

Fig. 2 presents the XRD graphs of pure PLA, PLA-20Ak, PLA-40Ak composites and akermanite powder. The graph of pure PLA consisted of a broad peak at the range of 10–25°, thus indicating its amorphous structure. Also, the XRD graph of akermanite powder consisted of sharp peaks at 2θ of 15.96, 21.02, 28.88, 31.09, 36.17, 44.39, 51.31 and 51.81°, which was in a good agreement with the standard ICDD card number 96-900-6941 (akermanite with the tetragonal crystal system).

XRD results of PLA-20Ak and PLA-40Ak indicated that the graphs consisted of a broad peak related to the amorphous PLA and sharp peaks of akermanite. The height of akermanite sharp peaks was increased with raising the akermanite content of the composite.

XRD graphs of the ultrasonic-assisted printed scaffold were not different from those of the untreated samples. This means ultrasonic vibrations did not affect the phases of samples.

### 3.3 Photograph of the printed scaffolds

**Fig. 3:**
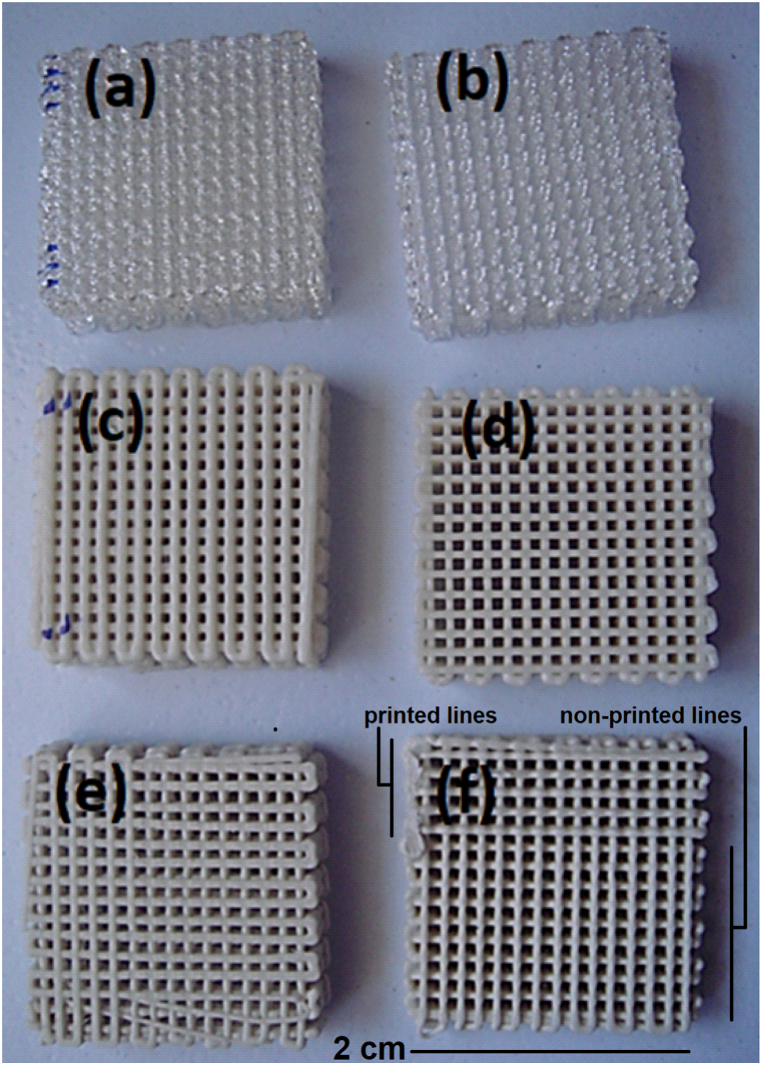
Top view of the printed scaffolds. (a and b): PLA scaffolds fabricated by ultrasonic-assisted 3D printing and conventional 3D printing; (c and d): PLA-20Ak scaffolds fabricated by ultrasonic-assisted 3D printing and conventional 3D printing; (e and f): PLA-40Ak scaffolds fabricated by ultrasonic-assisted 3D printing and conventional 3D printing. The samples have dimensions of 20 × 20 × 5 mm.

Fig. 3 presents the top view of the printed scaffolds. Fig. 3(a) and (b) also display the morphology of the pure PLA fabricated by ultrasonic-assisted 3D printing and conventional 3D printing, respectively. No significant difference was observed between them. Fig. 3(c) and (d) present the morphology of PLA-20Ak fabricated by ultrasonic-assisted 3D printing and conventional 3D printing, respectively. It seemed that the struts of the ultrasonic-assisted 3D-printed scaffold were thicker than those of the conventional 3D-printed scaffold, which might be due to more material extrusion. It could also be seen that ultrasonic vibration resulted in facile material extrusion and more material extrusion from the nozzle, because of the reduction in the viscosity of the molten composite [13,48].

Fig. 3(e) and (f) present the morphology of PLA-40Ak fabricated by ultrasonic-assisted 3D printing and conventional 3D printing, respectively. The ultrasonic-assisted 3D-printed scaffold (Fig. 3(e)) was completely printed and finished, but the conventional 3D-printed scaffold (Fig. 3(f)) was not completely printed; during printing, nozzle clogging happened.

### 3.4 Micrographs of 3D-printed scaffolds

**Fig. 4:**
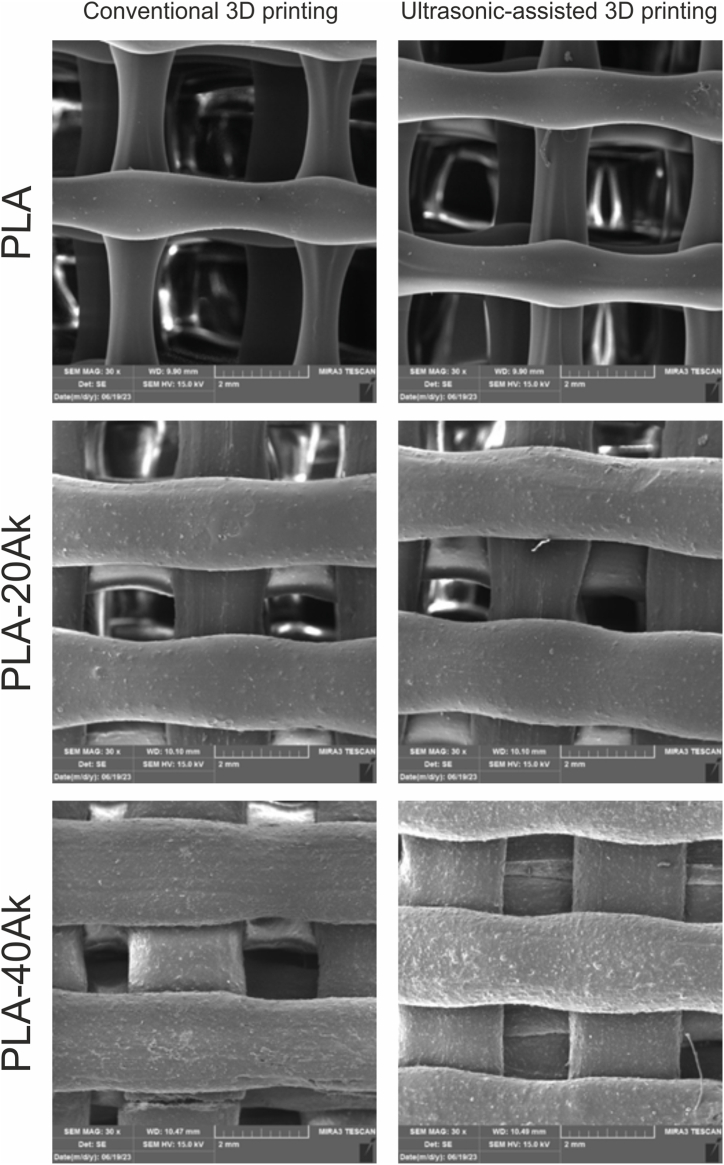
The SEM images (micrographs) of the surface of 3D-printed scaffolds (magnification: 30×).

Fig. 4 presents the micrograph of the surface of 3D-printed scaffolds. The micrograph of PLA scaffolds indicated that the struts of conventional 3D-printed PLA scaffolds were not uniform and their thickness at the junctions was thicker than that in other sections. Meanwhile, the struts of the ultrasonic-assisted 3D-printed PLA scaffold were more uniform in thickness. It seemed, therefore, that ultrasonic-assisted 3D printing resulted in more uniform extrusion of the materials from the nozzle, leading to the formation of uniform struts. Also, in both conventional 3D-printed and ultrasonic-assisted 3D-printed scaffolds, the pores were interconnected and uniform in geometry.

According to Fig. 4, it seemed that, during the 3D printing of PLA-20Ak and PLA-40Ak scaffolds, sonication was not effective enough and no significant difference could be observed between conventional 3D printing and ultrasonic-assisted 3D printing. Of course, during the printing of PLA-20Ak and PLA-40Ak scaffolds, more pneumatic pressure was required for material extrusion, as compared to PLA 3D printing, because of the higher viscosity of the molten composite.

During the conventional 3D printing of PLA-40Ak composite scaffold, nozzle clogging happened several times, and 3D printing was not successfully done to the last layer. But ultrasonic-assisted 3D printing of the PLA-40Ak composite scaffold was performed easily without nozzle clogging. This is since ultrasonic vibration can reduce the friction coefficient of akermanite particles and molten polymer or decrease the molten composite viscosity [13,48].

**Fig. 5:**
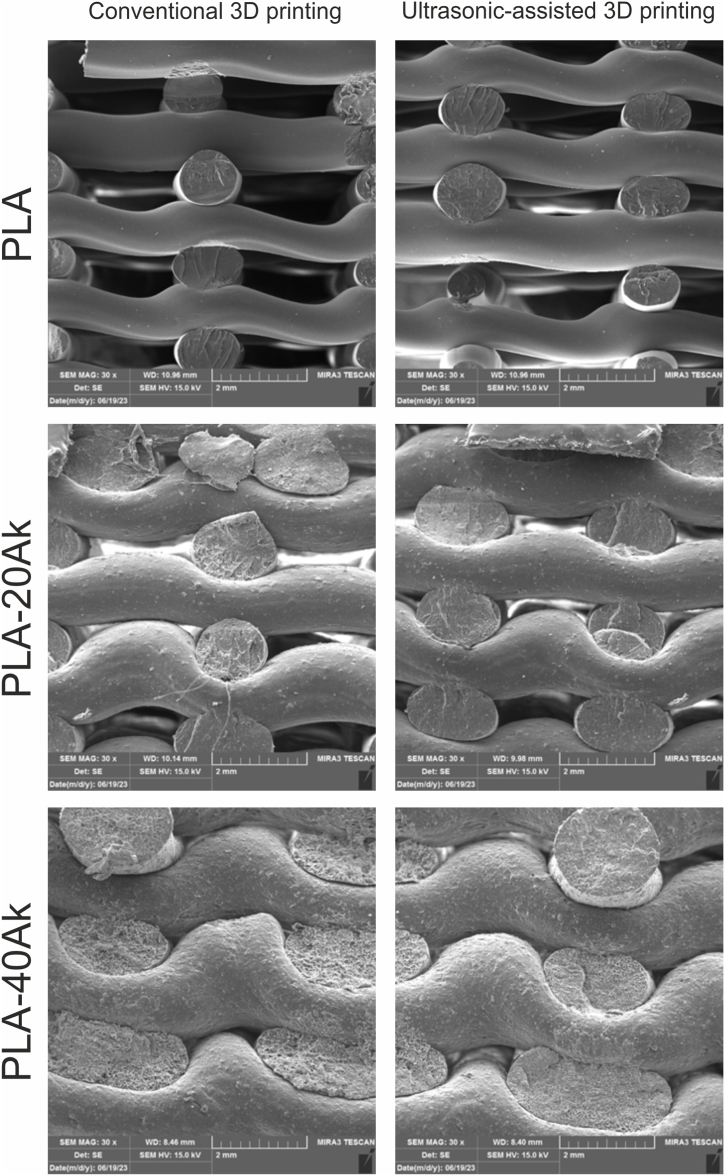
The SEM images (micrographs) of the cross-section of the 3D-printed scaffolds.

To investigate the effect of sonication on 3D printing, the photomicrograph of the cross-section of the samples was taken using SEM. The results are presented in Fig. 5. As can be seen, the pores were vertically interconnected. The struts with the raster angles of 90 and 0 were well-locked to each other, resulting in improved mechanical properties. Also, it was evident that the core of struts was rigid and without porosity. The fracture surface of struts became rougher by increasing the akermanite content of the composite. For example, the fracture surface of the struts of the pure PLA was smooth.

In general, three strategies can be considered for the boundary conditions of the vibrating system, including horn, nozzle and melt; the first one assumes that the horn tip is screwed or welded to the nozzle, and the triple assembly of the horn, nozzle and melt is considered a unified vibrating system whose natural frequency should be 35 kHz. In the second one, the horn tip is screwed or welded to the nozzle (similar to the first strategy), but only the double set of the horn and nozzle is considered as an integrated vibrational system with a natural frequency of 35 kHz. In the third one, only the horn is designed with the same natural frequency of 35 kHz. Anyway, adding ultrasonic vibrations to the 3D printer should be done in such a way that it can concentrate the vibration energy only on the melt coming out of the nozzle to apply mechanical vibrations to it with the maximum amplitude.

The first strategy means forcing the whole triple assembly to vibrate resonantly. This reduces the energy density of mechanical vibrations, as well as the amplitude of nozzle tip vibrations.

Considering free boundary condition for any lateral side of an object in modal analysis leads to antinode vibration creation in the desired face and consequently, the maximum vibration amplitude at the interface of two objects and further reduction of their friction and adhesion, which can be desirable [18]⸵ for example: in nozzle interface (integrated with the horn) and the melt in the second strategy. But the nozzle must also be screwed to the printer's extruder, which can change the mechanical stiffness of the vibrating system [20,21], ultimately leading to the release of ultrasonic waves to the printer's structure, as also observed in the first strategy. So, in this research, the third strategy was chosen; the horn tip was not connected to the nozzle, only touching the outer wall of the nozzle, which caused the nozzle and the melt inside to vibrate simultaneously. In this method, the harmonic impacts applied to the outer wall of the nozzle could lead to the vibration of the nozzle and the melt exiting the nozzle, without the stiffness of vibrational system being affected by any interacted objects [20,21] including the nozzle, the printer structure and the melt.

### 3.5 Compressive strength of scaffolds

**Fig. 6:**
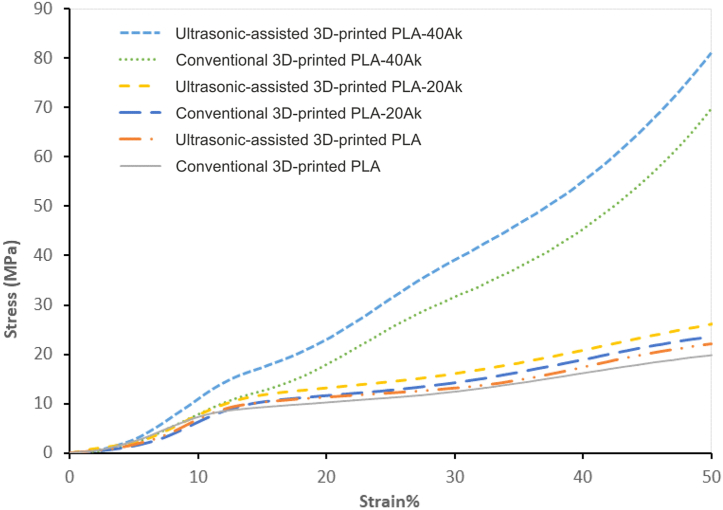
The stress-strain curve of a) conventional 3D-printed PLA, b) ultrasonic-assisted 3D-printed PLA, c) conventional 3D-printed PLA-20Ak, d) ultrasonic-assisted 3D-printed PLA-20Ak, e) conventional 3D-printed PLA-40Ak, and f) ultrasonic-assisted 3D-printed PLA-40A scaffolds.

The compressive stress-strain curves for the conventional 3D-printed PLA, ultrasonic-assisted 3D-printed PLA, conventional 3D-printed PLA-20Ak, ultrasonic-assisted 3D-printed PLA-20Ak, conventional 3D-printed PLA-40Ak, and ultrasonic-assisted3D-printed PLA-40A scaffolds are presented in Fig. 6. All curves showed distinct regions of elastic and plastic zones of deformation. The slope of the curve at the elastic zone could be considered as the elastic modulus of the scaffolds, and the algebraic mean of stress at the strain of 20–30 % or 20–40 % was taken as the compressive strength of them. It was, therefore, evident that the compressive strength and elastic modulus was increased by raising the akermanite content of the scaffold, or by applying ultrasonic vibration.

**Fig. 7:**
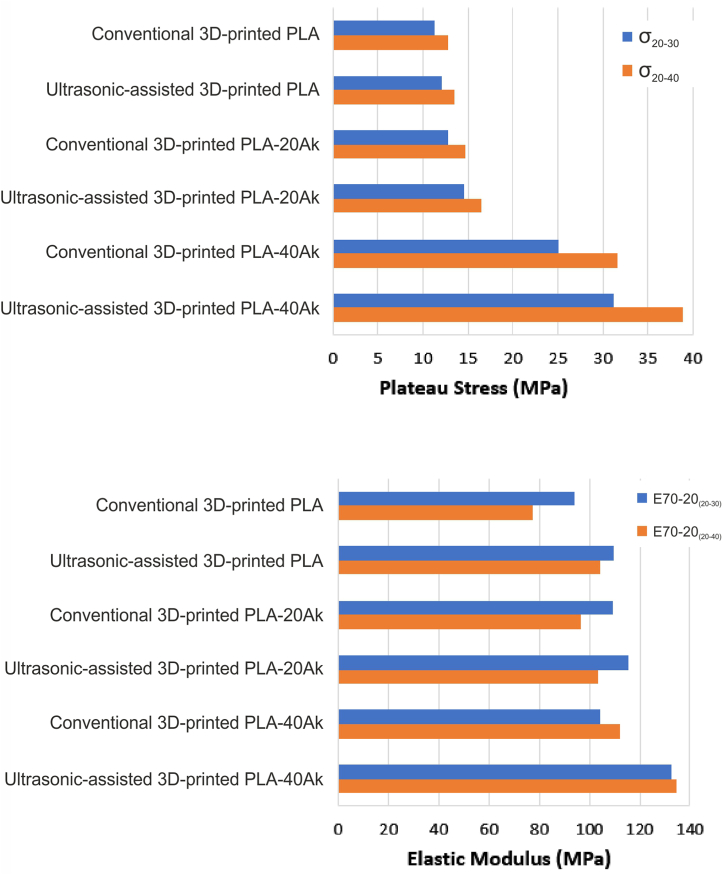
The plateau stress and elastic modulus of the scaffolds.

The values of elastic modulus and plateau (compressive) strength of the scaffolds are presented in Fig. 7 and Table 1. The elastic modulus of conventional 3D-printed PLA, ultrasonic-assisted 3D-printed PLA, conventional 3D-printed PLA-20Ak, ultrasonic-assisted 3D-printed PLA-20Ak, conventional 3D-printed PLA-40Ak, and ultrasonic-assisted 3D-printed PLA-40A scaffolds was 93.91, 109.8, 108.99, 115.56, 104.47, and 132.46 MPa, respectively. Also, the compressive strength (plateau stress at the strain of 20–30 %, σ_20-30_) of conventional 3D-printed PLA, ultrasonic-assisted 3D-printed PLA, conventional 3D-printed PLA-20Ak, ultrasonic-assisted 3D-printed PLA-20Ak, conventional 3D-printed PLA-40Ak, and ultrasonic-assisted 3D-printed PLA-40A scaffolds was 11.26, 12.15, 12.85, 14.56, 25.14, and 31.17 MPa, respectively. It was, therefore, evident that the elastic modulus and compressive strength of the scaffolds were raised by increasing the akermanite content or by applying ultrasonic vibration to the nozzle during 3D printing.

**Table 1:** The values of elastic modulus and plateau (compressive) strength of the scaffolds.

Scaffold	Conventional 3D-printed PLA	Ultrasonic-assisted 3D-printed PLA	Conventional 3D-printed PLA-20Ak	Ultrasonic-assisted 3D-printed PLA-20Ak	Conventional 3D-printed PLA-40Ak	Ultrasonic-assisted 3D-printed PLA-40A
The elastic modulus (E70-20 _(__20-30__)_, MPa)	93.91	109.8	108.99	115.56	104.47	132.46
The elastic modulus (E70-20 _(20-__4__0)_, MPa)	77.44	104.14	96.54	103.43	112.23	134.42
The plateau (compressive) strength (σ_20-30_, MPa)	11.26	12.15	12.85	14.56	25.14	31.17
The plateau (compressive) strength (σ_20-40_, MPa)	12.75	13.53	14.68	16.48	31.57	38.90

Meanwhile, the compressive strength (plateau stress at the strain of 20–40 %, σ_20-40_) of conventional 3D-printed PLA, ultrasonic-assisted 3D-printed PLA, conventional 3D-printed PLA-20Ak, ultrasonic-assisted 3D-printed PLA-20Ak, conventional 3D-printed PLA-40Ak, and ultrasonic-assisted 3D-printed PLA-40A scaffolds was 12.75, 13.53, 14.68, 16.48, 31.57, and 38.90 MPa, respectively. No matter whether the plateau stress was calculated at the strain of 20–30 or at a strain of 20–40, it was evident that the mechanical properties of scaffolds could be improved by increasing the akermanite content of them, or by applying ultrasonic vibration. Akermanite is a bioceramic harder than PLA, so its addition to PLA can result in increased strength. Additionally, applying ultrasonic treatment to the PLA-akermanite composite can lead to the better distribution of akermanite particles and a more homogeneous composite formation. Moreover, ultrasonic vibration can remove gas porosity from the molten polymer and create more rigid struts. Both factors can improve the mechanical properties of ultrasonically treated 3D-printed PLA-akermanite composites.

## 4 Conclusions

In this study, the effects of ultrasonic vibration on the printability and mechanical properties of 3D-printed PLA-Akermanite composite scaffolds were investigated. Ultrasonic vibration was found to inhibit nozzle clogging during the extrusion-based 3D printing of composites containing high akermanite nanoparticle loadings up to 40 wt%. Conventional 3D printing resulted in nozzle clogging when printing the PLA-40 wt%Ak composite, thus limiting the achievable nanoparticle content. In contrast, ultrasonic-assisted 3D printing enabled the successful fabrication of scaffolds with 40 wt% akermanite reinforcement without any nozzle clogging issues.

Characterization of the printed scaffolds showed that ultrasonic vibration did not alter the crystal phases of the composites. However, mechanical testing revealed that both the elastic modulus and compressive strength of the scaffolds were significantly improved by increasing the akermanite content. Additionally, applying ultrasonic vibration during printing further enhanced the mechanical properties. Scaffolds printed with ultrasonic assistance exhibited elastic modulus and compressive strength was increased by 25–27 %, as compared to their conventionally printed counterparts.

The results also demonstrated that ultrasonic vibration could be a highly effective approach for overcoming nozzle clogging during extrusion-based 3D printing of composites containing high viscosities or a high amount of nanoparticles. This novel ultrasonic-assisted printing method opens up new possibilities for fabricating scaffolds with optimized mechanical performance for tissue engineering applications by allowing higher nanoparticle loadings to be achieved. With further development, this technique shows promise for advancing extrusion 3D printing and expanding the range of materials that can be processed.

## CRediT authorship contribution statement

**Mohammad Khodaei:** Writing – review & editing, Methodology, Investigation, Conceptualization. **Hamed Razavi:** Writing – review & editing, Writing – original draft, Validation, Supervision, Investigation. **Hamed Nosrati:** Writing – review & editing, Writing – original draft.

## Ethical approval

No experiments on human or animal subjects were performed in this study.

## Data availability

All data that support the findings of this study are included within the article.

## Funding

This research did not receive any specific grant from any funding agencies in the public, commercial, or not-for-profit sectors.

## Declaration of competing interest

The authors declare that they have no known competing financial interests or personal relationships that could have appeared to influence the work reported in this paper.
